# Children and youth’s perceptions of mental health—a scoping review of qualitative studies

**DOI:** 10.1186/s12888-023-05169-x

**Published:** 2023-09-14

**Authors:** Linda Beckman, Sven Hassler, Lisa Hellström

**Affiliations:** 1 Department of Health Service, Management and Policy, University of Florida, 1125, Central Dr. 32610, Gainesville, FL USA; 2 Department of Public Health Science, Karlstad University, Universitetsgatan 2, 651 88 Karlstad, Sweden; 3 Department of School Development and Leadership, Malmö University, 211 19 Malmö, Sweden

**Keywords:** Mental health, Children, Youth, Perceptions, Public health, Scoping review

## Abstract

**Background:**

Recent research indicates that understanding how children and youth perceive mental health, how it is manifests, and where the line between mental health issues and everyday challenges should be drawn, is complex and varied. Consequently, it is important to investigate how children and youth perceive and communicate about mental health. With this in mind, our goal is to synthesize the literature on how children and youth (ages 10—25) perceive and conceptualize mental health.

**Methods:**

We conducted a preliminary search to identify the keywords, employing a search strategy across electronic databases including Medline, Scopus, CINAHL, PsychInfo, Sociological abstracts and Google Scholar. The search encompassed the period from September 20, 2021, to September 30, 2021. This effort yielded 11 eligible studies. Our scoping review was conducted in accordance with the PRISMA-ScR Checklist.

**Results:**

As various aspects of uncertainty in understanding of mental health have emerged, the results indicate the importance of establishing a shared language concerning mental health. This is essential for clarifying the distinctions between everyday challenges and issues that require treatment.

**Conclusion:**

We require a language that can direct children, parents, school personnel and professionals toward appropriate support and aid in formulating health interventions. Additionally, it holds significance to promote an understanding of the positive aspects of mental health. This emphasis should extend to the competence development of school personnel, enabling them to integrate insights about mental well-being into routine interactions with young individuals. This approach could empower children and youth to acquire the understanding that mental health is not a static condition but rather something that can be enhanced or, at the very least, maintained.

**Supplementary Information:**

The online version contains supplementary material available at 10.1186/s12888-023-05169-x.

## Introduction

In Western society, the prevalence of mental health issues, such as depression and anxiety [1], as well as recurring psychosomatic health complaints [2], has increased from the 1980s and 2000s. However, whether these changes in adolescent mental health are actual trends or influenced by alterations in how adolescents perceive, talk about, and report their mental well-being remains ambiguous [1]. Despite an increase in self-reported mental health problems, levels of mental well-being have remained stable, and severe psychiatric diagnoses have not significantly risen [3, 4]. Recent research indicates that understanding how children and youth grasp mental health, its manifestations, and the demarcation between mental health issues and everyday challenges is intricate and diverse. Wickström and Kvist Lindholm [5] show that problems such as feeling low and nervous are considered deep-seated issues among some adolescents, while others refer to them as everyday challenges. Meanwhile, adolescents in Hellström and Beckman [6] describe mental health problems as something mainstream, experienced by everyone at some point. Furthermore, Hermann et al. [7] point out that adolescents can distinguish between positive health and mental health problems. This indicates their understanding of the complexity and holistic nature of mental health and mental health issues. It is plausible that misunderstandings and devaluations of mental health and illness concepts may increase self-reported mental health problems and provide contradictory results when the understanding of mental health is studied. In a previous review on how children and young people perceive the concept of “health,” four major themes have been suggested: health practices, not being sick, feeling good, and being able to do the desired and required activities [8]. In a study involving 8–11 year olds, children framed both biomedical and holistic perspectives of health [9]. Regarding the concept of “illness,” themes such as somatic feeling states, functional and affective states [10, 11], as well as processes of contagion and contamination, have emerged [9]. Older age strongly predicts nuances in conceptualizations of health and illness [10–12].

As the current definitions of mental health and mental illness do not seem to have been successful in guiding how these concepts are perceived, literature has emphasized the importance of understanding individuals’ ideas of health and illness [9, 13]. The World Health Organization (WHO) broadly defines mental health as a *state of well-being in which the individual realizes his or her abilities, can cope with the normal stresses of life, work productively and fruitfully and make a contribution to his or her* community [14] capturing only positive aspects. According to The American Psychology Association [15], mental illness includes several conditions with varying severity and duration, from milder and transient disorders to long-term conditions affecting daily function. The term can thus cover everything from mild anxiety or depression to severe psychiatric conditions that should be treated by healthcare professionals. As a guide for individual experience, such a definition becomes insufficient in distinguishing mental illness from ordinary emotional expressions. According to the Swedish National Board of Health and Welfare et al. [16], *mental health* works as an umbrella term for both *mental well-being* and *mental illness*: *Mental well-being* is about being able to handle life's difficulties, feeling satisfied with life, having good social relationships, as well as being able to feel pleasure, desire, and happiness. *Mental illness* includes both *mild to moderate mental health problems* and *psychiatric conditions*. *Mild to moderate mental health problems* are common and are often reactions to events or situations in life, e.g., worry, feeling low, and sleep difficulties.

It has been argued that increased knowledge of the nature of mental illness can help individuals to cope with the situation and improve their well-being. Increased knowledge about mental illness, how to prevent mental illness and help-seeking behavior has been conceptualized as “mental health literacy” (MHL) [17], a construct that has emerged from “health literacy” [18]. Previous literature supports the idea that positive MHL is associated with mental well-being among adolescents [19]. Conversely, studies point out that low levels of MHL are associated with depression [20]. Some gender differences have been acknowledged in adolescents, with boys scoring lower than girls on MHL measures [20] and a social gradient including a positive relationship between MHL and perceived good financial position [19] or a higher socio-economic status [21].

While MHL stresses knowledge about signs and treatment of mental illness [22], the concern from a social constructivist approach would be the conceptualization of mental illness and how it is shaped by society and the thoughts, feelings, and actions of its members [23]. Studies on the social construction of anxiety and depression through media discourses have shown that language is at the heart of these processes, and that language both constructs the world as people perceive it but also forms the conditions under which an experience is likely to be construed [24, 25]. Considering experience as linguistically inflected, the constructionist approach offers an analytical tool to understand the conceptualization of mental illness and to distinguish mental illness from everyday challenges. The essence of mental health is therefore suggested to be psychological constructions identified through how adolescents and society at large perceive, talk about, and report mental health and how that, in turn, feeds a continuous process of conceptual re-construction or adaptation [26]. Considering experience as linguistically inflected, the constructionist approach could then offer an analytical tool to understand the potential influence of everyday challenges in the conceptualization of mental health.

Research investigating how children and youth perceive and communicate mental health is essential to understand the current rise of reported mental health problems [5]. Health promotion initiatives are more likely to be successful if they take people’s understanding, beliefs, and concerns into account [27, 28]. As far as we know, no review has mapped the literature to explore children’s and youths’ perceptions of mental health and mental illness. Based on previous literature, age, gender, and socioeconomic status seem to influence children's and youths’ knowledge and experiences of mental health [10–12]; therefore, we aim to analyze these perspectives too. From a social constructivist perspective, experience is linguistically inflected [26]; hence illuminating the conditions under which a perception of health is formed is of interest.

Therefore, we aim to study the literature on how children and youth (ages 10—25) perceive and conceptualize mental health, and the specific research questions are:

- What aspects are most salient in children’s and youths’ perceptions of mental health?
- What concepts do children and youth associate with mental health?
- In what way are children's and youth’s perceptions of mental health dependent on gender, age, and socioeconomic factors?

## Methods

### Literature search

**Fig. 1:**
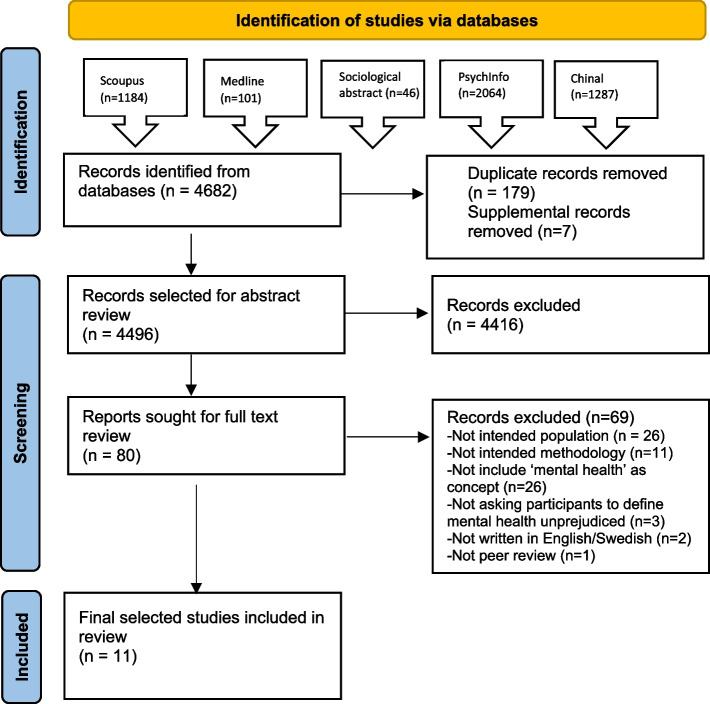
PRISMA flow diagram outlining the search process

A scoping review is a review that aims to provide a snapshot of the research that is published within a specific subject area. The purpose is to offer an overview and, on a more comprehensive level, to distinguish central themes compared to a systematic review. We chose to conduct a scoping review since our aim was to clarify the key concepts of mental health in the literature and to identify specific characteristics and concepts surrounding mental health [29, 30]. Our scoping review was conducted following the PRISMA-ScR Checklist [31]. Two authors (L.B and L.H) searched and screened the eligible articles. In the first step, titles and abstracts were screened. If the study included relevant data, the full article was read to determine if it met the eligibility criteria. Articles were excluded if they did not fulfill all the eligibility criteria. Any uncertainties were discussed among L.B. and L.H., and the third author, S.H., and were carefully assessed before making an inclusion or exclusion decision. The software Picoportal was employed for data management. Figure 1 illustrates a flowchart of data inclusion.

### Eligibility criteria

#### Population

We incorporated studies involving children and youth aged 10 to 25 years. This age range was chosen to encompass early puberty through young adulthood, a significant developmental period for young individuals in terms of comprehending mental health. Participants were required not to have undergone interviews due to chronic illness, learning disabilities (e.g., mental health linked to a cancer diagnosis), or immigrant status.

#### Context

Studies conducted in clinical settings were excluded. For the purpose of comparing results under similar conditions, we specifically opted for studies carried out in Western countries**.**

#### Concepts

Given that this review adopts a moderately constructionist approach, intentionally allowing for the exploration of how both young participants and society in general perceive and discuss mental health and how this process contributes to ongoing conceptual re-construction, the emphasis was placed on identifying articles in which participants themselves defined or attributed meaning to mental health and related concepts like mental illness. The criterion of selecting studies adopting an inductive approach to capture the perspectives of the young participants resulted in the exclusion of numerous studies that more overtly applied established concepts to young respondents [32].

#### Information sources

We utilized electronic databases and reached out to study authors if the article was not accessible online. Peer-reviewed articles were exclusively included, thereby excluding conference abstracts due to their perceived lack of relevance in addressing the review questions. Only research in English was taken into account. Publication years across all periods were encompassed in the search.

### Search strategy

Studies concerning children’s and youths’ perceptions of mental health were published across a range of scientific journals, such as those within psychiatry, psychology, social work, education, and mental health. Therefore, several databases were taken into account, including Medline, Scopus, CINAHL, PsychInfo, Sociological abstracts, and Google Scholar, spanning from inception on September 20, 2021 to September 30, 2021. We involved a university librarian from the start in the search process. The combinations of search terms are displayed in Table 1.

**Table 1:** Search terms

Search Terms
**Block 1**:**Target group:** adolescent* OR schoolchild* OR child* OR young OR student*
**Block 2: Mental health:** mental health OR wellbeing OR psychosomatic OR depression OR anxiety OR mental ill-health OR resilience OR coping strateg* OR stress OR distress OR sleepiness OR psychosocial problem* OR internalizing OR externalizing OR mood OR recreation OR self-esteem OR quality of life OR life satisfaction OR positive affect OR mindfulness OR self-efficacy OR psychological health OR life skill*
**Block 3:** perception* OR think* OR talk* OR understanding OR perspective* OR attitude*
**Block 4: Methods:** Personal Narratives OR qualitative OR interview* OR focus groups OR thematic analysis

### Quality assessment

We employed the Quality methods for the development of National Institute for Health Care Excellence (NICE) public health guidance [33] to evaluate the quality of the studies included. The checklist is based on checklists from Spencer et al. [34], Public Health Resource Unit (PHRU) [26, 35], and the North Thames Research Appraisal Group (NTRAG) [36] (Refer to S2 for checklist). Eight studies were assigned two plusses, and three studies received one plus. The studies with lower grades generally lacked sufficient descriptions of the researcher’s role, context reporting, and ethical reporting. No study was excluded in this stage.

### Data extraction and analysis

We employed a data extraction form that encompassed several key characteristics, including author(s), year, journal, country, details about method/design, participants and socioeconomics, aim, and main results (Table 2). The collected data were analyzed and synthesized using the thematic synthesis approach of Thomas and Harden [37]. This approach encompassed all text categorized as 'results' or 'findings' in study reports – which sometimes included abstracts, although the presentation wasn’t always consistent throughout the text. The size of the study reports ranged from a few sentences to a single page. The synthesis occurred through three interrelated stages that partially overlapped: coding of the findings from primary studies on a line-by-line basis, organization of these 'free codes' into interconnected areas to construct 'descriptive' themes, and the formation of 'analytical' themes.

**Table 2:** Summary of included studies studying child and youth’s perceptions of mental health

Study, Year, Country (Reference)	Study Design	Participants and Socioeconomic status of Sample	Aim and Concepts Identifying Mental health Used	Results
Armstrong, C. et al., (2000). Young People's Perceptions of Mental Health. [28] Country: UK	Semi-structured individual and focus group interviews	*n* = 145, ages 12–14. Focus groups (*n* = 120, 17 groups) and individual interviews (*n* = 18), participants recruited from 4 schools SES: schools with rural (higher unemployment rates) or urban (more affluent) and mixed SES environments	To explore the attitudes and perceptions of a broad range of young people aged 12–14 towards positive mental health and mental illness Concepts used: mentally healthy and unhealthy, positive mental health	• The term mental health was not salient and understandings of it were often uncertain • Those who focused on the word "mentally" usually associated this with mental illness even though the question had been about mental health • Socioeconomic difference in how to understand mental health: Mainly young people from schools in the most deprived area and the rural area who experienced these difficulties with the term mentally healthy • For some young people, the notion of mental health was closely linked with the idea of normality, although no consensus about what normality meant
Roose, G.A. & John, M.A. (2003). A focus group investigation into young children’s understanding of mental health and their views on appropriate services for their age group. [38] Country: UK	Semi-structured focus group interviews Data collected: reported	*n* = 16, ages 10–11, 2 focus groups with 8 participants each were recruited from two local primary schools, chosen because of their different locations and willingness to take part in the study SES: not reported	To explore 10- and 11-year-old children’s understanding of the concept of mental health and their opinions regarding an appropriate service for their age group Concepts used: mental health	• Mental health was understood to be complex • Children’s understanding of mental health was considered by the authors as sophisticated and many behaviors were considered to be indications of a serious problem. For example, not being able to put unhappiness aside, inability to concentrate on schoolwork, unusual behavior such as a lot of crying from someone who doesn’t usually cry and lying to cover up the sadness. As was the failure of normal strategies, ‘cos if you try, like you do normally to make it go away and it doesn't • Mental health was considered to have different aspects, emotions, thoughts and behavior, for example, ‘mental health is really peace of mind and not your emotions overbalancing’
Johansson, A., et al., (2007). Adolescent Girls’ and Boys’ Perceptions of Mental Health. [39] Country: Sweden	Semi-structured interviews, individual or focus groups	*n* = 48, ages 13 and 16, participants recruited from 2 schools SES: Participants were from different cultural backgrounds (but no closer description of this was done by the authors) Concepts used: mental health	To analyze the concept of mental health from the perspective of adolescent girls and boys and to describe what they regard as being important determinants of mental health	• The adolescents perceived mental health as an emotional experience (how you feel and what you think), where positive as well as negative health is part of the concept • Age differences seemed to be more important than gender in the perception of mental health by children • Younger girls and boys described their feelings more in relation to other people, friends and parents • The older girls expressed deeper negative emotions than the older boys and the younger children and could relate more easily to negative emotions than to positive emotions. Positive as well as negative emotions were divided between internal and relational emotions. The internal positive emotions were feeling of being happy, harmony, of being a good person and of having good self-confidence. The internal negative emotions were a feeling of being unhappy, of lack of meaning and hope, of being stressed and of having negative self-confidence • Older participants related mental health mostly to themselves
Landstedt E., et al., 2009). Understanding adolescent mental health: The influence of social processes, doing gender and gendered power relations [40] Country: Sweden	Semi-structured focus group interviews	*n* = 29 focus groups with 3–8 participants each age 16–19. Participants were recruited from schools in six towns of various sizes in rural and urban areas. The total sample varied in terms of age, socioeconomic and demographic characteristics with the goal of obtaining maximum variation SES: schools in six towns of various sizes in rural and urban areas, representing educational programmes, is.theoretical and vocational	To explore what 16- to 19-year-old students perceive as significant for adolescent mental health and, apply a gender analysis to the findings Concepts used: mental health	• Mental health was understood as an emotional experience and described as ‘how you feel’ in terms of self-esteem, stress, confidence and experiences of humiliation • Mental health was mainly associated with negative aspects, distress or illness • The participants emphasized that mental well-being, to a large extent, depends on trust, perceived respect, and appreciation for who one is—it’s important to be well treated, to be seen by others and to have somebody to trust • Joking represents both acts of bonding and assault (humiliation) and was expressed as sometimes contradictory and restraining • According to the participants, assault was associated with mental health in terms of humiliation, worry, anxiety, fear, stress, and insecurity • The adolescents considered a low degree of responsibility-taking as being positive for mental health which they exemplified as confidence, independence and feeling relaxed. A low degree of responsibility-taking was exemplified as ignoring or not responding to demands and things for which one is expected to take responsibility
Svirydzenka, N., et al., 2014). Schoolchildren's perspectives on the meaning of mental health. [41] Country: UK	Workshops with smaller groups. Some groups were asked to write on posters: What does the term mental health means? They concluded the workshops by asking the students to compare their results with the ‘bright futures’ definition of mental health	*n* = 218, age 13, participants recruited from 5 local schools and community colleges SES: not reported	To identify how schoolchildren defined mental health and how they thought they could keep themselves mentally healthy Concepts used: mental health, mental illness	• Four themes emerged: Personal attributes, disorder, personal management, and relationships • The “personal attributes” theme encompassed all the answers the students gave regarding themselves and their qualities that would play a part in defining mental health: “good” brain, emotional and physical functioning and development, high self-esteem, and a clear idea of who they are qualified as being “mentally healthy” • The “disorder” category included naming a mental health disorder • The “personal management” category included lifestyle choices (most common), being able to learn well, managing emotions, problem-solving and perceived or aspired control of the environment • The “relationships” category included being able to fit in with the world socially and having positive peer relationships were considered signifiers of good mental health
Chisholm, K., et al. 2016). Adolescent construction of mental illness: implication for engagement and treatment. [42] Country: UK	Participants were left to themselves in groups to discuss and define what they felt that 'mental health' might mean	*n* = 46, ages 11–18, participants recruited from 6 schools SES: Schools were selected to be broadly representative of Birmingham, UK	To develop a preliminary model of how adolescents perceive mental illness and construct their understanding of mental health Concepts used: mental health and mental illness	• Conflicting experiences and perceptions of mental illness. Participants discussed Stereotypes and Extreme symptoms of mental illness, but also displayed an insightful and empathetic understanding of the negative impact of stress, based on their own reality and experiences • Emotions of fear and anxiety were associated with both perceptions • Conflicting picture of mental illness. Distinctions were drawn; between ‘them’ and ‘us’, between ‘born with it’ and ‘developed’, and between ‘crazy’ and ‘diagnosed’ • Those with mental disorders were considered somehow ‘not normal’ or ‘different’ by participants • Participants in the older age groups interpreted mental health and illness as a continuum, rather than just focusing on the extreme examples. Older participants also talked about mental health in terms of positive emotions • Participants drew strong links between the term mental illness and derogatory terms such as “crazy”, but were less stigmatizing regarding individual diagnoses
Perre et al., (2016). Australian University Students’ Perceptions of Mental Illness: A Qualitative Study. [37] Country: Australia	Semi-structured interviews	*n* = 10, ages 19—24, participants recruited from one university. 9 out of 10 participants were girls SES: A mix of Australian-born and non- Australian born participants Concepts used: mentally healthy, mental health, mental illness	To explore young people’s perceptions of common mental health issues, predominantly depression and anxiety, alongside possible experiences of stigma	• Personal attributes in relation to the term ‘mentally healthy’, as ‘energy’ ‘confidence’ ‘happiness’, balance’, thinking logically, in-tuned with oneself • Mental health was linked with rationality • Participants expressed a sense of confusion surrounding diagnostic labeling of mental health issues and mental illness and held mixed opinions on the purpose of clinical diagnoses • Perceptions of depression varied amongst the participants, from those who aligned it with psychosis, to those who related it to simply ‘feeling down’. Most participants commented on depression as being a concern when it occurred frequently as opposed to transient feelings of sadness. Length of time that individuals had felt depressed and particularly loss of interest in socializing, were both seen as adequate measures for determining the difference between ‘feeling down’ and ‘clinical depression’ • Since young people have generally experienced the sensation of being anxious, nervous and apprehensive in their everyday lives, anxiety was harder to define in terms of it being an illness
Laidlaw, A., et al., (2016). Understanding undergraduate student perceptions of mental health, mental well-being and help-seeking behavior. [43] Country: UK	Semi-structured individual interviews	*n* = 20, ages 18–22, participants recruited from one university, and 5 different subject areas (psychology, biology, physics or English) SES: not reported	To improve their understanding of student perceptions of mental health, mental well-being and help-seeking behavior for difficulties in these areas Concepts used: mental health, mental wellbeing, mental illness	• Three different perceptions emerged in participants’ understanding of the terms mental health and mental well-being • One perspective considered mental health distinctly different from, and more clinical than, mental well-being. Participants with this perception commonly referred to mental health as ‘serious’, ‘psychiatric’, whereas mental well-being was described as ‘feeling happy, confident, able to function/cope, feeling secure’ • Another view included these concepts as a continuum with mental health at one end of the continuum and mental well-being at the other. Students in this group tended to perceive mental health as a more severe clinical mental illness whilst mental well-being was the absence of such illness • Participants also viewed the terms mental health and mental well-being as being the same thing, although they often appeared uncertain in this view • Participants' understanding of mental well-being and mental health fits with the dual factor model with the clinical mental health dimension perceived as being separate from the more everyday mental well-being dimension. Moreover, mental well-being issues are related to aspects of confidence and the ability to cope with life's demands
Teng, E., et al., (2017). Crying wolf? Australian adolescents’ perceptions of the ambiguity of visible indicators of mental health and authenticity of mental illness. [27] Country: Australia	Individual semi-structured interviews	*n* = 16, ages 12–18, participants recruited from 4 schools that were part of the school-based program MindMatters. All participants were Anglo-European Australians SES: Not reported	To explore how adolescents speak about mental health and illness Concepts used: mental health, mental wellbeing, mental illness	• Expressed uncertainty surrounding mental health and illness concepts and terminology • Negative aspects of mental health included notions of illness, such as psychological distress, traumatic experience, mental disorder, pessimism, and learning disabilities • Positive interpretations of mental health included notions of well-being, such as self-esteem, happiness, optimism and resilience, social support, and physical health but also some personality traits such as extraversion and intelligence • Some responses appeared to be more neutral, for example, referring to “thoughts and emotions” with no emphasis on either positive or negative aspects of mental health. Other neutral aspects included general ‘vibe’ • Mental illness included strong negative expressions • Depression and bipolar disorder were the mental illnesses mentioned most often • Expressed confusion over whether mental health was a positive or negative concept, interpreting the term mental health to refer to notions of knowledge and awareness • Authenticity—difficulties in trying to discern between those with good or poor mental health. E.g., lack of “visible proof” of mental illness. Experiences of doubt about whether peers reporting mental illness were “pretending” or “exaggerating” symptoms
O'Reilly, M. et al. (2018). Is social media bad for mental health and wellbeing? Exploring the perspectives of adolescents. [44] Country: UK	Semi-structured focus groups interviews	*n* = 54, age 11–18 years, 6 focus groups from schools in two UK cities (Leicester and London) SES: The sample was considered to reflect a broad diversity of socioeconomic and ethnic backgrounds (but no closer description of this was done by the authors)	To address what adolescents think of social media and its relevance to mental health and emotional wellbeing Concepts used: mental health, mental wellbeing, mental illness	• Many of the adolescents were unable to define mental health clearly, often confusing it with mental ill health. Others simply stated that they did not understand the term • This was particularly the case for younger participants who generally framed it as things that are happening in your brain or in terms of specific conditions like schizophrenia or simply not knowing: “I don’t understand what mental health is”. For example, they did not believe that mental health could be positive, and others asked the moderators for clarification
Molenaar, A. et al. (2020). Language of Health of Young Australian Adults: A Qualitative Exploration of Perceptions of Health, Wellbeing and Health Promotion via Online Conversations. [45] Country: Australia	Online conversations over social media	*n* = 166, ages 18–24, Participants were recruited by an Australian Market & Social Research Society-certified field house from three different International Organization for Standardization accredited panels	To provide formative information to inform future more hypothesis-driven research Concepts used: mental health, wellbeing, mental illness	• Mental health was highlighted as an important aspect of health, often due to being personally affected by mental health issues • The majority of participants in these analyses believed they were at their optimal health status and were therefore not largely concerned about illness or disease prevention but rather a more holistic view of health

## Results

The objective of this scoping review has been to investigate the literature concerning how children and youth (ages 10—25) conceptualize and perceive mental health. Based on the established inclusion- and exclusion criteria, a total of 11 articles were included representing the United Kingdom (*n* = 6), Australia (*n* = 3), and Sweden (*n* = 2) and were published between 2002 and 2020. Among these, two studies involved university students, while nine incorporated students from compulsory schools.

### Salient aspects of children and youth’ perceptions of mental health

Based on the results of the included articles, salient aspects of children’s and youths’ understandings revealed uncertainties about mental health in various ways. This uncertainty emerged as conflicting perceptions, uncertainty about the concept of mental health, and uncertainty regarding where to distinguish between mild to moderate mental health problems and everyday stressors or challenges.

One uncertainty was associated with *conflicting perceptions* that mental health might be interpreted differently among children and youths, depending on whether it relates to their *own* mental health or *someone else's* mental health status. Chisholm et al. [42] presented this as distinctions being made between ‘them and us’ and between ‘being born with it’. Mental health and mental illness were perceived as a continuum that rather developed’, and distinctions were drawn between ‘crazy’ and ‘diagnosed.’ Participants established strong associations between the term mental illness and derogatory terms like ‘crazy,’ linking extreme symptoms of mental illness with others. However, their attitude was less stigmatizing when it came to individual diagnoses, reflecting a more insightful and empathetic understanding of the adverse impacts of stress based on their personal realities and experiences. Despite the initial reactions reflecting negative stereotypes, further discussion revealed that this did not accurately represent a deeper comprehension of mental health and mental illness.

There was also uncertainty about *the concept of mental health*, as it was not always clearly understood among the participating youth. Some participants were unable to define mental health, often confusing it with mental illness [28]. Others simply stated that they did not understand the term, as in O’Reilly [44]. Additionally, uncertainty was expressed regarding whether mental health was a positive or negative concept [27, 28, 40, 44], and participants associated mental health with mental illness despite being asked about mental health [28]. One quote from a grade 9 student illustrates this: “*Interviewer:* Can mental health be positive as well? Informant: No, it’s mental” [44]. In Laidlaw et al. [46], with participants ranging from 18—22 years of age, most considered mental health distinctly different from and more clinical than mental well-being. However, Roose et al. [38], for example, the authors discovered a more multifaceted understanding of mental health, encompassing emotions, thoughts, and behavior. In Molenaar et al.[45], mental health was highlighted as a crucial aspect of health overall. In Chisholm et al. [42], the older age groups discussed mental health in a more positive sense when they considered themselves or people they knew, relating mental health to emotional well-being. Connected to the uncertainty in defining the concept of mental health was the uncertainty in identifying those with good or poor mental health. Due to the lack of visible proof, children and youths might doubt their peers’ reports of mental illness, wondering if they were pretending or exaggerating their symptoms [27].

A final uncertainty that emerged was difficulties *in drawing the line between psychiatric conditions and mild to moderate mental health problems and everyday stressors or challenges*. Perre et al. [43] described how the participants in their study were uncertain about the meaning of mental illness and mental health issues. While some linked depression to psychosis, others related it to simply ‘feeling down.’ However, most participants indicated that, in contrast to transient feelings of sadness, depression is a recurring concern. Furthermore, the duration of feeling depressed and particularly a loss of interest in socializing was seen as appropriate criteria for distinguishing between ‘feeling down’ and ‘clinical depression.’ Since feelings of anxiety, nervousness, and apprehension are common experiences among children and youth, defining anxiety as an illness as opposed to an everyday stressor was more challenging [43].

### Terms used to conceptualize mental health

When children and youth were asked about mental health, they sometimes used neutral terms such as thoughts and emotions or a general ‘vibe’ [27], and some described it as ‘peace of mind’ and being able to balance your emotions [38]. The notion of mental health was also found to be closely linked with rationality and the idea of normality, although, according to the young people, Armstrong et al. [28], there was no consensus about what ‘normal’ meant. Positive aspects of mental health were described by the participants as good self-esteem, confidence [40], happiness [39, 43], optimism, resilience, extraversion and intelligence [27], energy [43], balance, harmony [39, 43], good brain, emotional and physical functioning and development, and a clear idea of who they are [27, 41]. It also included a feeling of being a good person, feeling liked and loved by your parents, social support, and having people to talk with [27, 39], as well as being able to fit in with the world socially and positive peer relationships [41], according to the children and youths, mental health includes aspects related to individuals (individual factors) as well as to people in their surroundings (relationships). Regarding mental illness, participants defined it as stress and humiliation [40], psychological distress, traumatic experiences, mental disorders, pessimism, and learning disabilities [27]. Also, in contrast to the normality concept describing mental health, mental illness was described as somehow ‘not normal’ or ‘different’ in Chisholm et al. [42].

Depression and bipolar disorder were the most often mentioned mental illnesses [27]. The inability to balance emotions was seen as negative for mental health, for example, not being able to set aside unhappiness, lying to cover up sadness, and being unable to concentrate on schoolwork [38]. The understanding of mental illness also included feelings of fear and anxiety [42]. Other participants [46] indicated that mental health is distinctly different from, and more clinical than, mental well-being. In that sense, mental health was described using reinforcing terms such as ‘serious’ and ‘clinical,’ being more closely connected to mental illness, whereas mental well-being was described as the absence of illness, feeling happy, confident, being able to function and cope with life’s demands and feeling secure. Among younger participants, a more varied and vague understanding of mental health was shown, framing it as things happening in the brain or in terms of specific conditions like schizophrenia [44].

### Gender, age, socioeconomic status

#### Gender

Only one study had a gender theoretical perspective [40], but the focus of this perspective concerned gender differences in what influences mental health more than the conceptualization of mental health. According to Johansson et al.[39], older girls expressed deeper negative emotions (e.g., described feelings of lack of meaning and hope in various ways) than older boys and younger children.

#### Age

Several of the included studies noticed differences in age, where younger participants had difficulty understanding the concept of mental health [39, 44], while older participants used more words to explain it [39]. Furthermore, older participants seemed to view mental health and mental illness as a continuum, with mental illness at one end of the continuum and mental well-being at the other end [42, 46].

#### Socioeconomic status

The role of socioeconomic status was only discussed by Armstrong et al. [28], finding that young people from schools in the most deprived and rural areas experienced more difficulties defining the term mental health compared to those from a less deprived area.

## Discussion

This scoping review aimed to map children's and youth’s perceptions and conceptualizations of mental health. Our main findings indicate that the concept of mental health is surrounded by uncertainty. This raises the question of where this uncertainty stems from and what it symbolizes. From our perspective, this uncertainty can be understood from two angles. Firstly, the young participants in the different studies show no clear and common understanding of mental health; they express uncertainty about the meaning of the concept and where to draw the line between life experiences and psychiatric conditions. Secondly, uncertainty exists regarding how to apply these concepts in research, making it challenging to interpret and compare research results. The shift from a positivistic understanding of mental health as an objective condition to a more subjective inner experience has left the conceptualization open ranging from a pathological phenomenon to a normal and common human experience [47]. A dilemma that results in a lack of reliability that mirrors the elusive nature of the concept of mental health from both a respondent and a scientific perspective.

“Happy” was commonly used to describe mental health, whereas "unhappy" was used to describe mental illness. The meaning of happiness for mental health has been acknowledged in the literature, and according to Layard et al. [48], mental illness is one of the main causes of unhappiness, and happiness is the ultimate goal in human life. Layard et al. [48] suggest that schools and workplaces need to raise more awareness of mental health and strive to improve happiness to promote mental health and prevent mental illness. On the other hand, being able to experience and express different emotions could also be considered a part of mental health. The notion of normality also surfaced in some studies [38], understanding mental health as being emotionally balanced or normal or that mental illness was not normal [42]. To consider mental illness in terms of social norms and behavior followed with the sociological alternative to the medical model that was introduced in the sixties portraying mental illness more as socially unacceptable behavior that is successfully labeled by others as being deviant. Although our results did not indicate any perceptions of what ‘normal’ meant [28], one crucial starting point to the understanding of mental health among adolescents should be to delineate what constitutes normal functioning [23]. Children and youths’ understanding of mental illness seems to a large extent, to be on the same continuum as a normality rather than representing a medicalization of deviant behavior and a disjuncture with normality [49].

Concerning gender, it seemed that girls had an easier time conceptualizing mental health than boys. This could be due to the fact that girls mature verbally faster than boys [50], but also that girls, to a larger extent, share feelings and problems together compared to boys [51]. However, according to Johansson et al. [39], the differences in conceptualizations of mental health seem to be more age-related than gender-related. This could be due to the fact that older children have a more complex view of mental health compared to younger children.. Not surprisingly, the older the children and youth were, the more complex the ability to conceptualize mental health becomes. Only one study reported socioeconomic differences in conceptualizations of mental health [28]. This could be linked to mental health literacy (MHL) [18], i.e., knowledge about mental illness, how to prevent mental illness, and help-seeking behavior. Research has shown that disadvantaged social and socioeconomic conditions are associated with low MHL, that is, people with low SES tends to know less about symptoms and prevalence of different mental health problems [19, 21]. The perception and conceptualizations of mental health are, as we consider, strongly related to knowledge and beliefs about mental health, and according to von dem Knesebeck et al. [52] linked primarily to SES through level of education.

Chisholm et al. [42] found that the initial reactions from participants related to negative stereotypes, but further discussion revealed that the participants had more refined knowledge than at first glance. This illuminates the importance of talking to children and helping them verbalize their feelings, in many respects complex and diversified understanding of mental health. It is plausible that misunderstandings and devaluations of mental health and mental illness may increase self-reported mental health problems [5], as well as decrease them, preventing children and youth from seeking help. Therefore, increased knowledge of the nature of mental health can help individual cope with the situations and improve their mental well-being. Finding ways to incorporate discussions about mental well-being, mental health, and mental illness in schools could be the first step to decreasing the existing uncertainties about mental health. Experiencing feelings of sadness, anger, or upset from time to time is a natural part of life, and these emotions are not harmful and do not necessarily indicate mental illness [5, 6]. Adolescents may have an understanding of the complexity of mental health despite using simplified language but may need guidance on how to communicate their feelings and how to manage everyday challenges and normal strains in life [7].

With the aim of gaining a better understanding of how mental health is perceived among children and youth, this study has highlighted the concept’s uncertainty. Children and youth reveal a variety of understandings, from diagnoses of serious mental illnesses such as schizophrenia to moods and different types of behaviors. Is there only one way of understanding mental health, and is it reasonable to believe that we can reach a consensus? Judging by the questions asked, researchers also seem to have different ideas on what to incorporate into the concept of mental health — the researchers behind the present study included. The difficulties in differentiating challenges being part of everyday life with mental health issues need to be paid closer attention to and seems to be symptomatic with the lack of clarity of the concepts.

A constructivist approach would argue that the language of mental health has changed over time and thus influence how adolescents, as well as society at large, perceive, talk about, and report their mental health [26]. The re-construction or adaptation of concepts could explain why children and youth re struggling with the meaning of mental health and that mental health often is used interchangeably with mental illness. Mental health, rather than being an umbrella term, then represents a continuum with a positive and a negative end, at least among older adolescents. But as mental health according to this review also incorporates subjective expressions of moods and feelings, the reconstruction seems to have shaped it into a multidimensional concept, representing a horizontal continuum of positive and negative mental health and a vertical continuum of positive and negative well-being, similar to the health cross by Tudor [53] referred to in Laidlaw et al. [46] A multidimensional understanding of mental health constructs also incorporates evidence from interventions aimed at reducing mental health stigma among adolescents, where attitudes and beliefs as well as emotional responses towards mental health are targeted [54].

The contextual understanding of mental health, whether it is perceived in positive terms or negative, started with doctors and psychiatrists viewing it as representing a deviation from the normal. A perspective that has long been challenged by health workers, academics and professionals wanting to communicate mental health as a positive concept, as a resource to be promoted and supported. In order to find a common ground for communicating all aspects and dimensions of mental health and its conceptual constituents, it is suggested that we first must understand the subjective meaning ascribed to the use of the term [26]. This line of thought follows a social-constructionist approach viewing mental health as a concept that has transitioned from representing objective mental descriptions of conditions to personal subjective experiences. Shifting from being conceptualized as a pathological phenomenon to a normal and common human experience [47]. That a common understanding of mental health can be challenged by the healthcare services tradition and regulation for using diagnosis has been shown in a study of adolescents’ perspectives on shared decision-making in mental healthcare [55]. A practice perceived as labeling by the adolescents, indicating that steps towards a common understanding of mental health needs to be taken from several directions [55]. In a constructionist investigation to distinguish everyday challenges from mental health problems, instead of asking the question, “What is mental health?” we should perhaps ask, “How is the word ‘mental health’ used, and in what context and type of mental health episode?” [26]. This is an area for future studies to explore.

### Methodological considerations

The first limitation we want to acknowledge, as for any scoping review, is that the results are limited by the search terms included in the database searches. However, by conducting the searches with the help of an experienced librarian we have taken precautions to make the searches as inclusive as possible. The second limitation concerns the lack of homogeneous, or any results at all, according to different age groups, gender, socioeconomic status, and year when the study was conducted. It is well understood that age is a significant determinant in an individual’s conceptualization of more abstract phenomena such as mental health. Some of the studies approached only one age group but most included a wide age range, making it difficult to say anything specific about a particular age. Similar concerns are valid for gender. Regarding socioeconomic status, only one study reported this as a finding. However, this could be an outcome of the choice of methods we had — i.e., qualitative methods, where the aim seldom is to investigate differences between groups and the sample is often supposed to be a variety. It could also depend on the relatively small number of participants that are often used in focus groups of individual interviews- there are not enough participants to compare groups based on gender or socioeconomic status. Finally, we chose studies from countries that could be viewed as having similar development and perspective on mental health among adolescents. Despite this, cultural differences likely account for many youths’ conceptualizations of mental health. According to Meldahl et al. [56], adolescents’ perspectives on mental health are affected by a range of factors related to cultural identity, such as ethnicity, race, peer and family influence, religious and political views, for example. We would also like to add organizational cultures, such as the culture of the school and how schools work with mental health and related concepts [56].

## Conclusions and implications

Based on our results, we argue that there is a need to establish a common language for discussing mental health. This common language would enable better communication between adults and children and youth, ensuring that the content of the words used to describe mental health is unambiguous and clear. In this endeavor, it is essential to actively listen to the voices of children and youth, as their perspectives will provide us with clearer understanding of the experiences of being young in today’s world. Another way to develop a common language around mental health is through mental health education. A common language based on children’s and youth’s perspectives can guide school personnel, professionals, and parents when discussing and planning health interventions and mental health education. Achieving a common understanding through mental health education of adults and youth could also help clarify the boundaries between everyday challenges and problems needing treatment. It is further important to raise awareness of the positive aspect of mental health—that is, knowledge of what makes us flourish mentally should be more clearly emphasized in teaching our children and youth about life. It should also be emphasized in competence development for school personnel so that we can incorporate knowledge about mental well-being in everyday meetings with children and youth. In that way, we could help children and youth develop knowledge that mental health could be improved or at least maintained and not a static condition.

### Supplementary Information

**Additional file 1.**

## Data Availability

All data generated or analyzed during this study are included in this published article [and its supplementary information files].
