# Supplementary material for: Children and youth’s perceptions of mental health—a scoping review of qualitative studies
Source: BMC Psychiatry. 2023 Sep 14;23:669. doi: 10.1186/s12888-023-05169-x (PMC10503183; doi:10.1186/s12888-023-05169-x)
Supplement: Supplementary file 1 — Additional file 1. [file 12888_2023_5169_MOESM1_ESM.pdf]

Appendix H Quality appraisal checklist – qualitative studies

There is considerable debate over what quality criteria should be used to assess qualitative studies. Quality in qualitative research can be assessed using the same broad concepts of validity (or trustworthiness) used for quantitative research, but these need to be put in a different contextual framework to take into account the aims of qualitative research.

This qualitative checklist[17] is designed for people with a basic understanding of qualitative research methodology, and is based on the broadly accepted principles that characterise qualitative research and which may affect its validity. The following notes provide suggestions for completing the checklist. A list of publications on qualitative research is provided at the end of these notes for further reading on this topic.

The studies covered by this checklist are studies which collect and analyse qualitative data, usually (but not exclusively) textual (written), spoken or observational data. Qualitative data are occasionally collected by structured questionnaires (for example, as thematically organised free text comments), but such data needs to be carefully scrutinised as it may not meet acceptable quality criteria for consideration as a qualitative study.

The checklist's questions are framed in such a way so that it can encompass the variety of ways qualitative research is conducted. Care must be taken to apply the checklist in a way that matches the research methodology.

Please note that the sub questions given as examples under each question are intended to highlight some of the key issues to be considered for that question. They are not intended to be exhaustive. Please add any additional considerations in the comments box.

Notes on the completion of the separate sections of the checklist are appended to it.

In some circumstances it may be necessary to analyse qualitative material using a different approach, where the goal will be to seek to extract underlying theories, propositions and principles from the data, rather than focusing on the quality of the study per se. This may be appropriate where the aim is to gain particular insights into social processes. Where developments of the processes of appraisal are required these will be discussed with the CPHE team.

© NICE 2012. All rights reserved.

Checklist

**Study identification:** Include author, title, reference, year of publication		
**Guidance topic:**	**Key research question/aim:**	
**Checklist completed by:**		
**Theoretical approach**		
**1. Is a qualitative approach appropriate?**<br>For example:<br>• Does the research question seek to understand processes or structures, or illuminate subjective experiences or meanings?<br>• Could a quantitative approach better have addressed the research question?	Appropriate<br>Inappropriate<br>Not sure	Comments:
**2. Is the study clear in what it seeks to do?**<br>For example:<br>• Is the purpose of the study discussed – aims/objectives/research question/s?<br>• Is there adequate/appropriate reference to the literature?<br>• Are underpinning values/assumptions/theory discussed?	Clear<br>Unclear<br>Mixed	Comments:
**Study design**		

© NICE 2012. All rights reserved.

**3. How defensible/rigorous is the research design/ methodology?**<br>For example:<br>• Is the design appropriate to the research question?<br>• Is a rationale given for using a qualitative approach?<br>• Are there clear accounts of the rationale/justification for the sampling, data collection and data analysis techniques used?<br>• Is the selection of cases/sampling strategy theoretically justified?	Defensible<br>Indefensible<br>Not sure	Comments:
Data collection		
**4. How well was the data collection carried out?**<br>For example:<br>• Are the data collection methods clearly described?<br>• Were the appropriate data collected to address the research question?<br>• Was the data collection and record keeping systematic?	Appropriately<br>Inappropriately<br>Not sure/ inadequately reported	Comments:
Trustworthiness		
**5. Is the role of the researcher clearly described?**<br>For example:<br>• Has the relationship between the researcher and the participants been adequately considered?<br>• Does the paper describe how the research was explained and presented to the participants?	Clearly described<br>Unclear<br>Not described	Comments:

© NICE 2012. All rights reserved.

**6. Is the context clearly described?**<br>For example:<br>• Are the characteristics of the participants and settings clearly defined?<br>• Were observations made in a sufficient variety of circumstances<br>• Was context bias considered	Clear<br>Unclear<br>Not sure	Comments:
**7. Were the methods reliable?**<br>For example:<br>• Was data collected by more than 1 method?<br>• Is there justification for triangulation, or for not triangulating?<br>• Do the methods investigate what they claim to?	Reliable<br>Unreliable<br>Not sure	Comments:
Analysis		
**8. Is the data analysis sufficiently rigorous?**<br>For example:<br>• Is the procedure explicit – i.e. is it clear how the data was analysed to arrive at the results?<br>• How systematic is the analysis, is the procedure reliable/dependable?<br>• Is it clear how the themes and concepts were derived from the data?	Rigorous<br>Not rigorous<br>Not sure/not reported	Comments:

© NICE 2012. All rights reserved.

<table>
<tr><td>9. Is the data 'rich'?
For example:
How well are the contexts of the data described?
Has the diversity of perspective and content been explored?
How well has the detail and depth been demonstrated?
Are responses compared and contrasted across groups/sites?</td><td>Rich
Poor
Not sure/not reported</td><td>Comments:</td></tr>
<tr><td>10. Is the analysis reliable?
For example:
Did more than 1 researcher theme and code transcripts/data?
If so, how were differences resolved?
Did participants feed back on the transcripts/data if possible and relevant?
Were negative/discrepant results addressed or ignored?</td><td>Reliable
Unreliable
Not sure/not reported</td><td>Comments:</td></tr>
<tr><td>11. Are the findings convincing?
For example:
Are the findings clearly presented?
Are the findings internally coherent?
Are extracts from the original data included?
Are the data appropriately referenced?
Is the reporting clear and coherent?</td><td>Convincing
Not convincing
Not sure</td><td>Comments:</td></tr>
<tr><td>12. Are the findings relevant to the aims of the study?</td><td>Relevant
Irrelevant
Partially relevant</td><td>Comments:</td></tr>
</table>

© NICE 2012. All rights reserved.

**13. Conclusions**<br>For example:<br>• How clear are the links between data, interpretation and conclusions?<br>• Are the conclusions plausible and coherent?<br>• Have alternative explanations been explored and discounted?<br>• Does this enhance understanding of the research topic?<br>• Are the implications of the research clearly defined?<br>Is there adequate discussion of any limitations encountered?	Adequate<br>Inadequate<br>Not sure	Comments:
Ethics		
**14. How clear and coherent is the reporting of ethics?**<br>For example:<br>• Have ethical issues been taken into consideration?<br>• Are they adequately discussed e.g. do they address consent and anonymity?<br>• Have the consequences of the research been considered i.e. raising expectations, changing behaviour?<br>• Was the study approved by an ethics committee?	Appropriate<br>Inappropriate<br>Not sure/not reported	Comments:
Overall assessment		
**As far as can be ascertained from the paper, how well was the study conducted? (see guidance notes)**	++<br>+<br>−	Comments:

Notes on the use of the qualitative studies checklist

Section 1: theoretical approach

This section deals with the underlying theory and principles applied to the research.

© NICE 2012. All rights reserved.

1. Is a qualitative approach appropriate?

A qualitative approach can be judged to be appropriate when the research sets out to investigate phenomena which are not easy to accurately quantify or measure, or where such measurement would be arbitrary and inexact. If clear numerical measures could reasonably have been put in place then consider whether a quantitative approach may have been more appropriate. This is because most qualitative research seeks to explain the meanings which social actors use in their everyday lives rather than the meanings which the researchers bring to the situation.

Qualitative research in public health commonly measures:

- personal/lives experiences (for example, of a condition, treatment, situation)
- processes (for example, action research, practitioner/patient views on the acceptability of using new technology)
- personal meanings (for example, about death, birth, disability)
- interactions/relationships (for example, the quality of the GP/patient relationship, the openness of a psychotherapeutic relationship)
- service evaluations (for example, what was good/bad about patients experiences of a smoking cessation group).

2. Is the study clear in what it seeks to do?

Qualitative research designs tend to be theory generative rather than theory testing; therefore it is unlikely that a research question will be found in the form of a hypothesis or null hypothesis in the way that you would expect in conventional quantitative research. This does not mean however that the paper should not set out early and clearly what it is that the study is investigating and what the parameters are for that. The research question should be set in context by the provision of an adequate summary of the background literature and of the study's underpinning values and assumptions.

Section 2: study design

Considers the robustness of the design of the research project.

© NICE 2012. All rights reserved.

3. How defensible is the research design?

There are a large number of qualitative methodologies, and a tendency in health to 'mix' aspects of different methodologies or to use a generic qualitative method. From a qualitative perspective, none of this compromises the quality of a study as long as:

- The research design captures appropriate data and has an appropriate plan of analysis for the subject under investigation. There should be a clear and reasonable justification for the methods chosen.
- The choice of sample and sampling method should be clearly set out, (ideally including any shortcomings of the sample) and should be reasonable. It is important to remember that sampling in qualitative research can be purposive and should not be random. Qualitative research is not experimental, does not purport to be generalisable, and therefore does not require a large or random sample. People are usually 'chosen' for qualitative research based on being key informers.

Section 3: data collection

4. How well was the data collection carried out?

Were the method of data collection the most appropriate given the aims of the research? Was the data collection robust, are there details of:

- how the data were collected?
- how the data were recorded and transcribed (if verbal data)?
- how the data were stored?
- what records were kept of the data collection?

Section 4: trustworthiness

Assessing the validity of qualitative research is very different from quantitative research. Qualitative research is much more focused on demonstrating the causes of bias rather than eliminating them, as a result it is good practice to include sections in the report about the reflexive position of the researcher (what was their 'part' in the research?), about the context in which the research was conducted, and about the reliability of the data themselves.

© NICE 2012. All rights reserved.

5. Is the role of the researcher clearly described?

The researcher should have considered their role in the research either as reader, interviewer, or observer for example. This is often referred to as 'reflexivity'. It is important that we can determine: a clear audit trail from respondent all the way through to reporting, why the author reported what they did report, and that we can follow the reasoning from the data to the final analysis or theory.

The 'status' of the researcher can profoundly affect the data, for example, a middle aged woman and a young adult male are likely to get different responses to questions about sexual activity if they interview a group of teenage boys. It is important to consider age, gender, ethnicity, 'insider' status (where the interviewer/researcher is part of the group being researched or has the same condition/illness, for example). The researcher can also profoundly influence the data by use of questions, opinions and judgments, so it is important to know what the researchers' position is in that regard and how the researcher introduced and talked about the research with the participants.

6. Is the context clearly described?

It is important when gauging the validity of qualitative data to engage with the data in a meaningful way, and to consider whether the data are plausible/realistic. To make an accurate assessment of this it is important to have information about the context of the research, not only in terms of the physical context – for example, youth club, GP surgery, gang headquarters, who else was there (discussion with parents present or discussion with peers present are likely to cause the participant to position himself very differently and thus to respond very differently) – but also in terms of feeling that the participants are described in enough detail that the reader can have some sort of insight into their life/situation. Any potential context bias should be considered.

7. Were the methods reliable?

It is important that the method used to collect the data is appropriate for the research question, and that the data generated map well onto the aims of the study. Ideally, more than 1 method should have been used to collect data, or there should be some other kind of system of comparison which allows the data to be compared. This is referred to as triangulation.

Section 5: analysis

Qualitative data analysis is very different from quantitative analysis. This does not mean that it should not be systematic and rigorous but systematicity and rigour require different methods of assessment.

© NICE 2012. All rights reserved.

8. Is the data analysis sufficiently rigorous?

The main way to assess this is by how clearly the analysis is reported and whether the analysis is approached systematically. There should be a clear and consistent method for coding and analysing data, and it should be clear how the coding and analytic strategies were derived. Above all, these must be reasonable in light of the evidence and the aims of the study. Transparency is the key to addressing the rigour of the analysis.

9. Are the data rich?

Qualitative researchers use the adjective 'rich' to describe data which is in-depth, convincing, compelling and detailed enough that the reader feels that they have achieved some level of insight into the research participants experience. It's also important to know the 'context' of the data, that is, where it came from, what prompted it and what it pertains to.

10. Is the analysis reliable?

The analysis of data can be made more reliable by setting checks in place. It is good practice to have sections of data coded by another researcher, or at least have a second researcher check the coding for consistency. Participants may also be allowed to verify the transcripts of their interview (or other data collection, if appropriate). Negative/discrepant results should always be highlighted and discussed.

11. Are the findings convincing?

In qualitative research, the reader should find the results of the research convincing, or credible. This means that the findings should be clearly presented and logically organised, that they should not contradict themselves without explanation or consideration and that they should be clear and coherent.

Extracts from original data should be included where possible to give a fuller sense of the findings, and these data should be appropriately referenced – although you would expect data to be anonymised, it still needs to be referenced in relevant ways, for example if gender differences were important then you would expect extracts to be marked male/female.

12–13. Relevance of findings and conclusions

These sections are self-explanatory.

© NICE 2012. All rights reserved.

Section 6: ethics

14. How clear and coherent is the reporting of ethics?

All qualitative research has ethical considerations and these should be considered within any research report. Ideally there should be a full discussion of ethics, although this is rare because of space limitations in peer-reviewed journals. If there are particularly fraught ethical issues raised by a particularly sensitive piece of research, then these should be discussed in enough detail that the reader is convinced that every care was taken to protect research participants.

Any research with human participants should be approved by a research ethics committee and this should be reported.

Section 7: overall assessment

15. Is the study relevant?

Does the study cast light on the review being undertaken?

16. How well was the study conducted?

Grade the study according to the list below:

++ All or most of the checklist criteria have been fulfilled, where they have not been fulfilled the conclusions are very unlikely to alter.

+ Some of the checklist criteria have been fulfilled, where they have not been fulfilled, or not adequately described, the conclusions are unlikely to alter.

– Few or no checklist criteria have been fulfilled and the conclusions are likely or very likely to alter.

---

[17] This checklist is based on checklists in:

Spencer L. Ritchie J, Lewis J et al. (2003) Quality in qualitative evaluation: a framework for assessing research evidence. London: Government Chief Social Researcher's Office

Public Health Resource Unit (2006) 10 questions to help you make sense of qualitative research [accessed 7 July 2008]

© NICE 2012. All rights reserved.

North Thames Research Appraisal Group (NTRAG): 1998 Critical review form for reading a paper describing qualitative research British Sociological Association (BSA)

© NICE 2012. All rights reserved.
